# Under-five mortality and maternal HIV status in Tanzania: analysis of trends between 2003 and 2012 using AIDS Indicator Survey data

**DOI:** 10.3402/gha.v9.31676

**Published:** 2016-06-20

**Authors:** Malachi Ochieng Arunda, Vikas Choudhry, Björn Ekman, Benedict Oppong Asamoah

**Affiliations:** 1 International Master Programme in Public Health, Faculty of Medicine, Lund University, CRC, Jan Waldenströms gata 35, 205 02 Malmö, Sweden; 2 Social Medicine and Global Health, Department of Clinical Sciences, Malmo, Lund University, Sweden

**Keywords:** under-five mortality, HIV, cross-sectional design, logistic regression, antiretroviral therapy, prevention of mother-to-child transmission

## Abstract

**Background:**

Mortality among children under five remains a significant health challenge across sub-Saharan Africa. HIV/AIDS is one of the leading contributors to the relatively slow decline in under-five mortality in this region. In Tanzania, HIV prevalence among under-five children is high and 90% of all infections are due to mother-to-child transmission.

**Objectives:**

The study aimed to examine the association between maternal HIV-positive status and under-five mortality in Tanzania. It also aimed to estimate the proportions and trends of under-five mortality attributable to maternal HIV/AIDS in Tanzania between 2003 and 2012.

**Design:**

Binomial logistic regression was used to analyze cross-sectional survey data from the Tanzania AIDS Indicator Surveys to examine the association between maternal HIV positivity and under-five mortality between 2003 and 2012.

**Results:**

After controlling for confounders, the adjusted odds ratios were 1.5 (95% CI 1.1–1.9) in 2003–2004, 4.6 (95% CI 2.7–7.8) in 2007–2008, and 2.4 (95% CI 1.2–4.6) in 2011–2012. The maternal HIV-attributable mortality risk percent of under-five children was 3.7 percent in 2003–2004, 11.3 percent in 2007–2008 and 5.6% in 2011–2012.

**Conclusion:**

Maternal HIV positivity is associated with under-five mortality in Tanzania, making maternal HIV serostatus a relevant determinant of whether a child will survive up to five years of age or not. The impact of maternal HIV/AIDS attributable mortality risk has a significant contribution to the overall under-five mortality in Tanzania. The continued monitoring of HIV and mortality trends is important for policy development and design of interventions.

## Introduction

HIV/AIDS continues to be one of the leading causes of death among children under the age of five in countries with high prevalence of HIV (1). Several cohort studies in sub-Saharan Africa (SSA) show that under-five mortality rates are higher among children born to HIV-positive mothers as compared to those born to HIV-negative mothers (2–6). Globally, more than 90% of all HIV infections among children are caused by vertical transmission from infected mothers (7) during gestation, delivery, postpartum, and during breastfeeding (8–13). One contributing factor to this spread of HIV among children is the relatively low rate at which mothers are tested for the disease during pregnancy. Demographic and health survey (DHS) reports from 2003 to 2012 indicate that more than 90% of the pregnant women in Tanzania had at least one antenatal care (ANC) visit by a skilled care provider (14). Overall estimates in SSA show that between 2003 and 2011, less than half of all expectant mothers were tested for HIV during their antenatal visits (14). However, in Tanzania, the proportion of pregnant women who were tested for HIV during ANC visits increased from 12.2% in 2003 through 43.3% in 2007 to 70.6% in 2010, among women who gave birth 2 years prior to the respective surveys (14).

Almost half of the 5.9 million global under-five deaths in 2015 occurred in SSA (15). In 2008, HIV accounted for 4% of all under-five mortality in SSA (16, 17), and the prevalence of HIV among women in SSA between 2009 and 2012 was 8.3% (18). A study by Newell et al. estimated that, at 2 years of age, about seven times more HIV-infected children in Africa (52.5%) would have died as compared to the uninfected ones (7.6%) (19). Another study in rural South Africa found that in 2012, four times more deaths occurred among infected children than uninfected ones between the ages of two-five years (6).

The lack of comprehensive interventions targeted at prevention of mother-to-child transmission (PMTCT) of HIV is one of the primary reasons for the high rates of under-five mortality in SSA (20). Further, indirect effects of HIV/AIDS such as maternal morbidity and death also contribute to under-five mortality, irrespective of the children's HIV status (2). In many SSA countries, strategies used for PMTCT are based on the revised World Health Organization (WHO) recommendations for the PMTCT of HIV for low- and middle-income countries (21). It outlines two approaches: 1) lifelong antiretroviral therapy (ART) for HIV-infected women and 2) antiretroviral prophylaxis to prevent transmission during pregnancy, delivery, and breastfeeding (21). The WHO also recommends the commencement of ART for HIV-positive pregnant women with CD4 count ≤350 cells/mm^3^ (21). Additionally, Tanzania's National Multi-Sectoral Strategic Framework III (2013–2017) is currently implementing and expanding its PMTCT programs. The Health Sector HIV and AIDS Strategic Plan III also aims to increase ART coverage for HIV-positive children to 70% by 2017 in an effort to reduce AIDS-attributable mortalities among children (22). However, challenges such as weak early infant diagnosis, ineffective PMTCT, shortage of drugs, and the fact that many women give birth at home have hindered both the assessment of the impact of MTCT (mother-to-child transmission) and the monitoring and evaluation of PMTCT intervention efforts (22).

Tanzania, among 21 SSA countries and India, forms part of the global plan priority countries. The aim of this global plan was to ‘reduce the number of children newly infected with HIV by 90%’ by the end of 2015 (23). Profound progress has been made by some of these global plan priority African countries (23). Between 2009 and 2013, Malawi registered 67% progress in reducing new HIV infections among children; Botswana, Ethiopia, Ghana, Mozambique, Namibia, South Africa, and Zimbabwe also achieved 50% or more progress (23). However, Tanzania registered comparatively slow progress – less than 50%, despite having increased the HIV/AIDS counselling and testing facilities by over 50% in the same period (23, 24).

A number of community-based studies in rural and Peri-urban areas in SSA countries including Tanzania have also found associations between under-five mortality and maternal HIV positivity (5, 6, 25, 26). A few health facility–based studies in Tanzania, both qualitative and quantitative, concentrated mainly on the challenges, infant-feeding practices of HIV-positive mothers, and the risks of mortality (27, 28). However, no peer-reviewed, nationwide study that focused on the associations between under-five mortality and maternal HIV positivity was identified. The aim of this study is to examine the association between maternal HIV-positive status and under-five mortality in Tanzania. The study also aims to estimate the proportions and trends of under-five mortality attributable to maternal HIV/AIDS in Tanzania between 2003 and 2012. The results are intended to add to the pool of evidence on MTCT, with implications for improving HIV/AIDS-related programs and enabling policy makers to look backwards and plan forward for effective PMTCT.

## Methods

### Study setting

With a population of about 45 million and a fertility rate of 5 in 2012 (29), Tanzania has one of the highest birthrates in the world (29). Agriculture is the main economic activity and about 80% of the population live in rural areas (30, 31). Twenty-eight percent of the population is poor (30). The sex ratio is roughly 1:1 (29), and in 2012 almost 16% of the population was below 5 years of age (30). Women of childbearing age (15–49) constitute about 23% of the population (30). Close to 30,000 babies are born every day in Tanzania (29). The MTCT rate of HIV among pregnant mothers who accessed any prophylaxis was 20% in 2010, while those who did not receive any treatment had an estimated transmission rate of 35% (17, 32). Challenges in the health system operations or socioeconomic and cultural hindrances have impeded intervention efforts (33, 34). Poor remuneration, shortage, and inadequate distribution of health workers are some of the challenges faced by the health system operations (34). Although the DHS 2013 comparative report indicated that the coverage for at least one ANC visit in Tanzania has been over 80% since 2003 (14), about 50% of all pregnant women did not give birth in health facilities in 2011 (35, 36).

### Study design and data sources

We used secondary data from the Tanzania AIDS Indicator Survey (AIS) datasets of the DHS program for the years 2003, 2007, and 2011. These are repeated cross-sectional surveys that collect a range of data on independent samples of women and children. The data sampling and collection method of the AIS involves random sampling of data across the whole country in a cross-sectional survey design. Moreover, the datasets are nationally representative. Information on reproduction, demographic characteristics, sexual activity, and HIV/AIDS are collected through individual interviews. Blood samples are taken from individuals aged 15–49 for testing. The biomarker survey (HIV test results) component of the AIS is coded and stored separately. Standardized DHS/AIS questionnaires and protocols are used, and the survey participants remain anonymous. Further details on data collection methods of the AIS can be obtained from the DHS/AIS methodology toolkits and field manuals (37, 38). The updated sampling frame and list of enumeration areas ensures that each survey covers the whole country without overlap (39).

We used three AIS datasets of the surveys conducted in 2003–2004, 2007–2008, and 2011–2012. The datasets were merged with the corresponding biomarker survey components (HIV test results).

### Variables

#### Outcome variable

*Under-five mortality*. This variable was defined as the death of a child before reaching 5 years of age at the time the respective surveys were conducted.

#### Predictor variable

*Maternal HIV-status*. Data on HIV were obtained through laboratory testing for HIV during the AIS and verbally through interviews about HIV testing during ANC visits of the most recent birth under 5 years.

### Maternal background and characteristics

The variables in this study constituted those variables that are hypothesized to be risk factors to under-five mortality based on existing evidence. For instance, low socioeconomic status leads to undernutrition among children, and poor water supply, especially in rural residency, results in infectious diseases such as diarrhea, both of which are leading causes of under-five deaths (40, 41). Further, poverty and lack of maternal education have been associated with under-five mortality (41). The independent variables constituted non-causal risk factors to both HIV infections and mortality and were considered as confounding factors in this study. The categorization of maternal age groups in this study was based on findings from other studies that associated certain maternal age ranges with the risk of under-five mortality. Children born to teenage and younger mothers and those born to older women (age 35+) are associated with greater risk of mortality as compared to those born to mothers between 25 and 34 years of age (42). A number of maternal health and child survival studies in SSA have used similar age classifications (18, 43). Studies have reported associations between poor household wealth status and under-five mortality (44). The DHS construction and categorization of wealth status in this study were based on the asset index of socioeconomic status (36). Detailed description and categorization of all the study variables are outlined in the summary of variables box below.

### Summary of variables

**Table:** 

Variable	Categorization	Description/composition
**Outcome variable**		
Under-five mortality	Yes (dead)	Died at age <5 years
	No (alive)	Alive at age ≥5 years
**Predictor variable**		
Maternal HIV status	HIV positive	
	HIV negative	
**Independent variables**		
Maternal education	Uneducated	No formal education
	Primary	≤9 years of education
	Secondary or higher	≥9 years of education
Marital status	Single	Never married, separated, widowed, or divorced and not living together
	Married	Married or cohabiting at the time of death
Maternal age (years)	15–24	
	25–34	
	35–49	
Place of residence	Rural	
	Urban	
Parity	Nulliparous	First-time child-bearers
	Para 1–3	1–3 births
	Para 4+	4+ births
Wealth status	Poor	Included poor and the poorest
	Middle	Middle income
	Wealthy	Wealthy and the wealthiest
Access to clean water	Yes	Piped, rain, protected sources
	No	Rivers, unprotected sources

*Parity* was defined as the number of times a woman has given birth to a fetus of 24 or more weeks gestation age (42).		

## Statistical methods

We conducted a Pearson's chi-square test of independence and association to examine the distribution of maternal and background characteristics according to mother's HIV status and under-five survival status. Binomial logistic regression analysis was used to determine odds ratios (ORs) after adjusting for possible confounders. Children with no known or suspected HIV exposure or no exposure were excluded in the regression analysis. Maternal HIV status was assessed as the main exposure variable. Independent/confounding variables were included in the regression analysis one at a time (stepwise) for each of the study periods while observing the changes in the odds of under-five deaths by maternal HIV status. The confounders included maternal age, place of residence, maternal education level, marital status, parity, wealth status, and drinking water source. Statistical significance was considered at *p*<0.05 (95% confidence level). We used SPSS version 22.0 statistical software (IBM, Armonk, New York, USA) for analysis and Microsoft Excel to generate graphical representation of HIV/AIDS-attributable under-five mortality.

### Estimation of HIV-attributable risk fraction and HIV population-attributable risk fraction

The HIV/AIDS-attributable mortality risk fraction (AF) and population-attributable mortality risk fraction (PAF) (in percentages) were calculated as the proportion of prevalent under-five death cases that could be avoided if the exposure (maternal HIV and transmission of HIV from mother to child) was eliminated. These were computed manually using Equations 1 and 2:

1 $$AF=\frac{\left(OR–1\right)}{OR}\text{*}100$$

Similarly,

2 $$PAF=Pe\text{*}AF=Pe\text{*}\left(\frac{OR-1}{OR}\right)\text{*}100$$

where OR is the adjusted odds ratio obtained from logistic regression analysis and *Pe* is the proportion of deaths that have the exposure.

## Results

Univariate analysis of all the variables in Table 1 shows that the proportions of under-five deaths in all the categories were generally higher in 2003–2004 as compared to the other survey periods. Almost half (48.9%) of all the under-five children born to HIV-positive mothers died in less than 5 years prior to the 2003–2004 survey. The proportions of these deaths decreased progressively in 2007–2008 and even further in 2011–2012. Similarly, the proportion of deaths in all other independent variable categories decreased progressively from 2003 to 2012 as shown in Table 1. The mothers’ mean age was between 27 and 30 years for all the survey periods. Table 2 indicates that more than 70% of the mothers lived in rural areas and over 87% of all the women were married. The proportion of HIV-positive mothers in the urban areas decreased by 10% from 2003 to 2008 and by less than 1% from 2007 to 2012. In contrast, the proportion of HIV-positive mothers in the rural areas rose by 10% from 2003 to 2008 and by less than 1% from 2007 to 2012.

**Table 1:** Distribution of background and maternal characteristics of under-five children, by under-five mortality status in Tanzania between 2003 and 2012 (95% confidence limit)

	2003–2004			2007–2008			2011–2012		
			
	Mortality, *N*=2,371			Mortality, *N*=4,625			Mortality, *N*=5,552		
			
Baseline characteristics	Yes (%)	No (%)	*p*	Yes (%)	No (%)	*p*	Yes (%)	No (%)	*p*
Maternal HIV status									
HIV positive	48.9	51.1	<0.01	11.6	88.4	<0.01	5.6	94.4	<0.01
HIV negative	32.0	68.0		3.5	96.5		2.5	97.5	
Maternal age^a^	(*N*=2,672)			(*N*=4,910)			(*N*=5,845)		
15–24	16.2	83.8	<0.01	4.0	96.0	0.2	3.1	96.9	0.50
25–34	36.9	63.1		3.4	96.6		2.4	97.6	
35–49	60.6	39.4		4.7	95.3		2.5	97.5	
Place of residence									
Rural	35.1	64.9	<0.01	3.7	96.3	0.03	2.5	97.5	0.1
Urban	23.4	76.6		5.3	94.7		3.4	96.6	
Education									
No education	43.0	57.0	<0.01	3.8	96.2	0.5	2.3	97.7	0.4
Primary	30.6	69.4		4.2	95.8		3.7	96.3	
Secondary	12.2	87.8		3	97		3.3	96.7	
Marital status									
Single	13.2	86.8	<0.01	5.3	94.7	0.3	2.9	97.1	0.8
Married	34.5	65.5		96.1	3.9		2.6	93.4	
Wealth status									
Poor	39.4	60.6	<0.01	3.5	96.5	0.3	2.7	97.3	<0.01
Middle	34.4	65.6		3.5	96.5		1.4	98.6	
Wealthy	23.1	76.9		4.5	95.5		3.3	96.7	
Parity									
Nulliparous	5.7	94.3	<0.01	4.7	95.3	0.5	3.4	96.4	0.2
Para 1–3	26.3	73.7		3.8	96.2		2.7	97.3	
Para 4+	62.8	27.6		3.8	96.2		2.2	97.8	
Clean water^a^									
No	36.3	63.7	<0.01	4.0	96.0	0.5	2.3	97.7	0.4
Yes	23.7	76.3		3.6	96.4		2.7	97.3	
Mean age^b^	27.6			29.4			29.5		

Statistical significance (*p*<0.05, two-sided).

a access to clean drinking water

b Represents maternal age at birth.

**Table 2:** Distribution of background characteristics of under-five children, by maternal HIV status in Tanzania between 2003 and 2012 (95% confidence limit)

	2003–2004 (*N*=2,371)			2007–2008 (*N*=4,625)			2011–2012 (*N*=5,551)		
			
Variable	HIV+	HIV−	*p*	HIV+	HIV−	*p*	HIV+	HIV−	*p*
Place of residence									
Urban	53 (40.5)	419 (18.7)	<0.01	67 (29.9)	720 (16.4)	<0.01	73 (29.1)	720 (16.4)	<0.01
Rural	78 (59.5)	1,821 (81.3)		157 (70.1)	3,681 (83.6)		178 (70.9)	3,681 (83.6)	
Education									
No education	19 (14.5)	516 (23.0)	<0.01	39 (17.4)	1,192 (27.1)	<0.01	40 (15.9)	1,186 (22.4)	<0.01
Primary	101 (77.1)	1,641 (73.3)		169 (75.5)	2,758 (62.7)		190 (75.7)	3,435 (64.8)	
Secondary/higher	11 (8.4)	83 (3.7)		16 (7.1)	451 (10.2)		21 (8.4)	680 (12.8)	
Maternal age									
15–24	43 (32.8)	895 (39.9)	0.1	67 (29.9)	1,313 (29.8)	0.5	53 (21.8)	1,189 (24.2)	0.04
25–34	69 (52.7)	969 (43.3)		104 (46.4)	1,908 (43.4)		133 (54.7)	2,284 (46.6)	
35–49	19 (14.5)	376 (16.8)		53 (23.7)	1,180 (26.8)		57 (23.5)	1,433 (29.2)	
Marital status									
Single	17 (13)	194 (8.7)	0.09	14 (6.2)	211 (4.8)	0.3	18 (7.9)	211 (4.8)	0.7
Married	144 (87)	2,046 (91.3)		210 (93.8)	4,190 (95.2)		210 (92.1)	4,190 (95.2)	
Wealth status									
Poor	41 (31.3)	1,044 (46.6)	<0.01	87 (52.1)	1,698 (47.1)	0.4	87 (34.7)	2,214 (41.8)	<0.01
Middle class	22 (16.8)	447 (20)		36 (21.6)	896 (24.9)		45 (17.9)	1,080 (20.4)	
Wealthy	68 (51.9)	749 (33.4)		44 (26.3)	1,011 (28.0)		119 (47.4)	2,007 (37.9)	
Clean water^a^									
No	35 (34.7)	1,108 (54.1)	<0.01	82 (39.8)	1,750 (42.0)	0.5	105 (47.7)	2,210 (48.0)	0.9
Yes	66 (65.3)	939 (45.9)		124 (60.2)	2,420 (58.0)		115 (52.3)	2,396 (52.0)	

X^2^*p* value – Pearson's chi-square test of independence. Statistically significant observation (*p*<0.05, two-sided).

a refers to drinking water.

The binomial logistic regression analysis in Table 3 shows that, after controlling for possible confounding variables, maternal HIV-positive status was significantly associated with under-five mortality in all the survey years. The risk of under-five mortality due to maternal HIV exposure varied from 2003 to 2012. The adjusted odds ratio (aOR) was lowest in 2003–2004, aOR 1.5 (95% CI 1.1–1.9), highest in 2007–2008, aOR 4.6 (95% CI 2.7–7.8), and lower in 2011–2012, aOR 2.4 (95% CI 1.2–4.6).

**Table 3:** Binomial logistic regression analysis of the association between under-five mortality and exposure to maternal HIV*-*positive status among Tanzanian children between 2003 and 2012; adjusted odds ratios (aOR*s*) (95% confidence interval)

	2003–2004 (*N*=2,371)		2007–2008 (*N*=4,625)		2011–2012 (*N*=5,552)	
			
Variables	aOR (95% CI)	*p*	aOR (95% CI)	*p*	aOR (95% CI)	*p*
Maternal HIV status						
HIV positive	1.5 (1.1–1.9)	<0.01	4.6 (2.7–7.8)	<0.01	2.4 (1.2–4.6)	<0.01
HIV negative	1.0		1.0		1.0	
Place of residence						
Rural	0.7 (0.5–0.9)	0.04	0.9 (0.5–1.6)	1.0	0.9 (0.5–1.6)	0.4
Urban	1.0		1.0		1.0	
Education level^a^						
No education	0.2 (0.1–0.6)	<0.01	0.6 (0.2–1.6)	0.2	1.2 (0.6–2.6)	0.3
Primary education	0.3 (0.1–1.0)	0.1	0.6 (0.2–1.6)	0.2	1.0 (0.5–1.8)	0.9
Marital status						
Single	0.8 (0.4–1.6)	0.4	0.6 (0.2–1.6)	0.3	0.6 (0.2–1.8)	0.5
Married	1.0		1.0		1.0	
Wealth status^b^						
Poor	0.8 (0.6–1.1)	0.1	1.6 (1.0–2.5)	0.04	1.1 (0.7–1.9)	0.6
Middle class	0.8 (0.5–1.1)	0.1	1.7 (0.9–2.8)	0.51	2.7 (1.2–5.7)	0.01
Maternal age^c^						
15–24	1.8 (1.3–2.5)	<0.01	0.9 (0.5–1.5)	0.5	0.8 (0.5–1.3)	0.4
35–49	0.6 (0.5–0.8)	<0.01	0.7 (0.5–1.2)	0.8	0.9 (0.5–1.5)	0.7
Parity^d^						
Para 1–3	0.4 (0.2–0.7)	<0.01	1.4 (0.8–2.5)	0.5	1.1 (0.6–2.1)	0.5
Para 4+	0.1 (0.03–0.14)	<0.01	1.4 (0.7–2.9)	0.7	1.3 (0.6–2.7)	0.5
Clean drinking water						
No	1.7 (1.3–2.1)	<0.01	1.4 (0.9–2.1)	0.7	0.7 (0.5–1.5)	0.6
Yes	1.0		1.0		1.0	

Adjusted for all variables in the table. Reference groups:

a secondary/higher education

b wealth,

c age group 25–34,

d nulliparous.

**Fig. 1:**
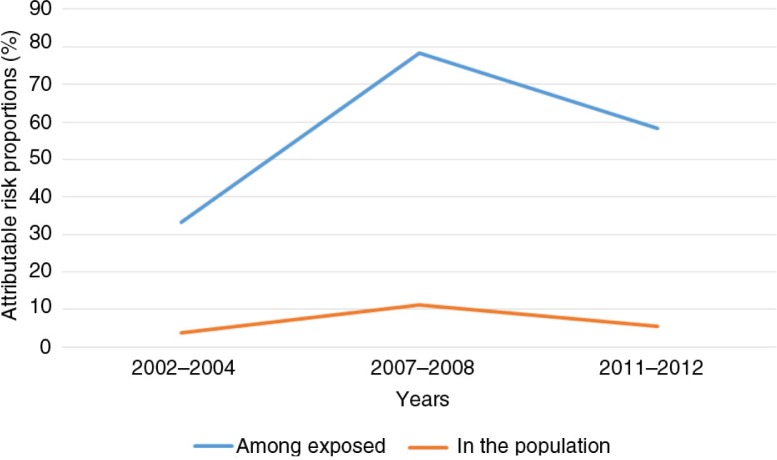
Trends of HIV/AIDS*-*attributable risk percent of under-five mortality between 2003 and 2012, among *children* exposed *to HIV* and in the entire Tanzanian population.

The AF and PAF were highest in 2007 and lowest in 2003 (Table 4). In 2003–2004, the attributable risk percent among the exposed children was 33.3%, and in the entire population it was 3.7%. About 78% of all HIV-exposed children died between 2005 and 2008, and these deaths were attributable to their mothers’ HIV-positive status. During the same period, about 11% of under-five deaths in Tanzania could be attributed to maternal HIV-positive status. Analysis of the most recent data in this study indicated that in 2011–2012, about 5.6% of under-five deaths in the Tanzanian population were attributable to maternal HIV-positive status. Results from 2011 to 2012 show that about 58% of all deaths among HIV-exposed children could be attributed to maternal HIV-positive status. Figure 1 shows the trends in mortality attributable to maternal HIV-positive status, with peaks in 2007–2008 among both those exposed to HIV and in the entire population. The slopes for both the rise and the decline of HIV-attributable under-five mortalities are more gradual in the entire population as compared to those among exposed children, which are relatively steep.

**Table 4:** HIV/AIDS-attributable mortality risk fraction among exposed under-five children and in the entire under-five population in Tanzania

	Survey year	Attributable risk fraction (%)
For children of infected mothers		
	2003–2004	33.3
	2007–2008	78.3
	2011–2012	58.3
In the entire population		
	2003–2004	3.7
	2007–2008	11.3
	2011–2012	5.6

In 2003–2004 (Table 3), the aOR showed that the maternal age group 15–24 and lack of clean drinking water were significantly associated with the risk of under-five mortality as shown by the aOR, *p*<0.05. Parity 1–3, para 4+, older women (35+), no education, and rural residency were protective against under-five mortality in 2003–2004. It was only in 2007–2008 that the under-five mortality risk among the poor was 1.6 times higher as compared to the wealthy and this was statistically significant. Similarly, it was only in 2011–2012 that the middle class wealth status had almost three times higher risk of under-five deaths as compared to the wealthy. All the other independent variables in 2007–2008 and 2011–2012 showed no significant associations with under-five mortality.

## Discussion

The overall odds of mortality adjusted for maternal age, parity, drinking water source, marital and wealth status, level of education, and place of residence were about 1.5–4.6 times higher for under-five children born to HIV-positive mothers as compared to those born to HIV-negative mothers between 2003 and 2012. The highest mortality risk (4.6 times higher) among under-five children born to HIV-infected mothers was observed in 2007–2008. One to two out of every three deaths among children under 5 years born to HIV-positive mothers could be attributed to their mothers’ HIV seropositive status, which represents 3.7–11.3% of under-five mortality in the entire Tanzanian population. A number of studies in Uganda, Malawi, and South Africa found similar statistically significant associations between maternal HIV positivity and under-five mortality (2–6).

The present study did not separately examine mortality among HIV-uninfected and HIV-infected children born to HIV-positive mothers. However, we sought to examine whether under-five mortality in Tanzania was significantly associated with maternal HIV positivity. Our findings indicated a strong association between maternal HIV and under-five mortality; therefore this study supports the continued access and provision of option B/B+ of the revised WHO guidelines that propose ARV prophylaxis during pregnancy, delivery, and postpartum during breastfeeding for PMTCT (21).

This study revealed a tripling of under-five mortality risk due to maternal HIV from 1.5 in 2003 to 4.6 in 2007–2008. These estimates are comparable with a rise in the prevalence of HIV among young women 20–24 years of age in Tanzania between 2003 and 2008 (33), where more than 23% of teenagers have begun childbearing (45). Our study thus considers inadequate the PMTCT programs or protocols that existed in Tanzania between 2003 and 2007. During this period, HIV-attributable deaths among under-five children also tripled. This could be further explained by the fact that it was only beginning in 2007 that ART was provided free of charge to children (46). Many poor families were hindered from accessing HIV testing and ART for children before 2007 due to unaffordable costs. Consequently, this led to higher HIV-attributable under-five deaths in 2007–2008. Lack of resources was also noted by Fiscus et al. as a profound hindrance to treatment (47). Comprehensive knowledge on HIV/AIDS among females aged 15–24 in Tanzania had also declined between 2003 and 2008 (22), thus fueling the infections and mortalities.

From a public health viewpoint and health policy evaluation perspective, our results detected obvious progress in reducing HIV-attributable mortality among under-five children in Tanzania between 2007 and 2012. Within 5 years of PMTCT interventions in Tanzania, under-five mortality attributed to maternal HIV decreased by half, from 11.3% in 2007 to 5.6% in 2012. These results concur with the 2009 aggregate findings of research in SSA countries (3.6%) and in Tanzania (5.0%) (17). Our 2011–2012 estimate of population attributable mortality risk is also comparable with the HIV prevalence of 5.6–6.0% among women in Tanzania in 2011 (48, 49). Furthermore, a comprehensive assessment of PMTCT programs in northern Tanzania indicated that MTCT of HIV decreased from 15.2% in 2008 to 3.1% in 2010 (50). We can therefore theoretically estimate that by scaling up the northern Tanzania PMTCT intervention programs nationwide to reach both rural and urban areas, the HIV-attributable under-five mortality risk in Tanzania could be reduced to near zero by 2017. This study suggests the need for continued public health funding of the current interventions in order to eliminate the HIV-attributable deaths among under-five children.

The variation in HIV-attributable mortality observed over the 10-year period in this study reveals that intervention gaps exist and that PMTCT has been a challenge for a long time. Similar studies have also revealed the existence of persistent challenges hindering PMTCT interventions (2–4). One of the challenges identified in 2013 by the Mitra Plus 2-year follow-up study among Tanzanian women was poor adherence to ART for PMTCT (51). Moreover, in many SSA countries, the PMTCT guidelines have largely focused on HIV-positive mothers tested during antenatal visits and less on HIV-negative mothers (52). In Zimbabwe it was noted that maternal infection during the postpartum period and the subsequent infection of babies during breastfeeding had received little attention (52). In this regard, a more proactive approach to expedite the decrease in HIV-attributable under-five mortality in Tanzania may need to involve community follow-up of almost 50% of all pregnant women, who currently give birth in homes (35) and miss out on HIV screening during delivery at the health facilities. This approach could also profoundly suppress the social stigma associated with HIV in communities, since every pregnant woman would receive HIV-related attention irrespective of their serostatus. Additionally, further research on the gaps of HIV infections among children such as seroconversion during pregnancy and subsequent infection of children ought to be investigated (53, 54).

The 3.7% HIV-attributable mortality of under-five children in 2003–2004 in this study could arguably be taken as an underestimate when compared with other study findings that reported an overall estimate of 10% in all of SSA in 2002 (55). However, the disparities can be accounted for by the fact that there have been larger variations in the impact of HIV across SSA regions. For example, in 2003, the HIV-attributable child mortality in Southern Africa was about 15.5%, while in Central Africa it was about 2.5% (20). Moreover, the prevalence of HIV also varies substantially across SSA countries (56).

Numerous studies analyzing the effect of sociodemographic and maternal factors on under-five mortality have already been conducted (41, 43, 57–59) and our findings partly concur with these studies. HIV/AIDS is also known to exacerbate sociodemographic conditions that indirectly lead to deaths. The 2003–2004 findings showed a significant association between lack of clean drinking water and under-five mortality. However, the significance in this association progressively disappeared in 2008 and 2012. This improvement could be explained by findings from UNICEF, which reported that, as the access to clean drinking water for children improved from 2003 to 2010, in both urban and rural Tanzania (60), the risks of mortality from waterborne diseases among children decreased (60). Continuous improvement in the lives of mothers and households through education and economic empowerment is an undebatable factor in the fight against HIV-attributable under-five mortality.

### Methodological consideration

The random sampling of data across the whole country is a key strength and partly a justification for the external validity of our study. However, the possibility of information bias during data collection through individual interviews cannot be completely ruled out, particularly with respect to recall on the part of participants. Our study does not confirm the causal association between under-five mortality and maternal HIV-positive status. This is because neither the children's HIV status nor the actual cause of death was ascertained medically. Further, our study could not confirm whether the mother became infected before or after the baby died, since many HIV test results were mainly obtained during the surveys. The under-five mortality (0–4 years) may be underreported. Other limitations include missing information on the exact age at death of the children, no sufficient data on the use of antiretroviral drugs among children, and maternal survival information.

## Conclusions

Maternal HIV-positive status is associated with under-five mortality in Tanzania and is one of the key predictors of under-five survival in Tanzania. It contributes a proportion of deaths to the overall under-five mortality. Continuous research to monitor trends and evaluate PMTCT programs will have a positive impact on reducing under-five mortality in Tanzania.

Tanzania lags behind in its efforts to achieve both global and national targets on reducing maternal HIV-attributable mortalities among under-five children. Best practices currently adopted are based on program evaluation reports that are insufficient due to weak infant diagnosis and limited information about births and under-five mortality among HIV-positive mothers. This study provides evidence-based national estimates of under-five mortality trends, thus enabling a more comprehensive nationwide evaluation and subsequent policy development towards the target.

## Ethical considerations

Confidentiality is safeguarded in DHS/AIS data. All identifiers were removed and the participants remained anonymous. Datasets are publicly accessible on request and permission to access, download, and store the datasets for this study was obtained in September 2015 from ORC Macro, Inc.
